# Supplementary material for: Health Risk Assessment of Dermal Exposure to Polycyclic Aromatic Hydrocarbons from the Use of Infant Diapers
Source: Int J Environ Res Public Health. 2022 Nov 10;19(22):14760. doi: 10.3390/ijerph192214760 (PMC9691111; doi:10.3390/ijerph192214760)
Supplement: Supplementary file 1 [file ijerph-19-14760-s001.zip › ijerph-1993247-supplementary.pdf]

Table S1 Concentration of polycyclic aromatic carbons (PAHs) in the diaper core of infant diapers from four brands.

PAHs (µg/kg)	Brand of diapers			
	18-01042	18-01046	18-01033	18-01051
Benzo[a]pyrene	< 0.48	0.66 ± 0.43	0.97 ± 0.63	0.31 ± 0.20
Dibenzo[a,h]anthracene	0.32 ± 0.29	< 0.27	1.4 ± 1.2	< 0.12
Benzo[g,h,i]perylene	0.48 ± 0.37	0.94 ± 0.75	1.5 ± 1.12	< 0.23
Naphthalene	10.1 ± 4.5	19.3 ± 8.5	12.5 ± 5.5	12.9 ± 8.7
Anthracene	< 1.47	< 2.18	< 2.0	< 0.85
Benzo[a]anthracene	< 0.57	0.66 ± 0.35	0.99 ± 0.46	0.21 ± 0.16
Indeno[1,2,3-cd]pyrene	< 0.40	0.79 ± 0.70	1.3 ± 1.1	< 0.27
Chrysene	< 0.22	0.63 ± 0.49	0.87 ± 0.47	< 0.15
Benzo[b]fluoranthene	< 0.56	< 0.75	< 0.87	< 0.30
Benzo[k]fluoranthene	< 0.36	0.69 ± 0.53	1.12 ± 0.84	< 0.24
Acenaphthene	0.45 ± 0,21	1.11 ± 0.51	0.76 ± 0.35	0.80 ± 0.37
Acenaphthylene	< 0.50	<0.72	<0.69	< 0.28
Phenanthrene	2.8 ± 3.3	4.6 ± 5.5	3.2 ± 3.9	3.7 ± 4.4
Fluoranthene	< 0.30	1.59 ± 0.71	1.20 ± 0.53	1.06 ± 0.47
Fluorene	0.96 ± 0.45	1.44 ± 0.69	1.61 ± 0.73	1.45 ± 0.66
Pyrene	< 0.58	0.93 ± 0.42	0.97 ± 0.43	0.85 ± 0.38

Table S2 Concentrations of polycyclic aromatic hydrocarbons (PAHs) in the top sheet (A), back sheet, (C) and fastening tapes (F) of infant diapers from 6 brands.

BLV-Nr.	18-01033-A	18-01033-C	18-01033-F
METAS-Nr.	200044712	200044713	200044711
Ace [ug/kg]	<4.63	<0.62	x
Acy [ug/kg]	7.35 +/-3.21	<0.53	8.54 +/-4.87
Ant [ug/kg]	6.05 +/-2.53	<0.50	x
BaA [ug/kg]	7.31 +/-3.29	<0.71	x
BaP [ug/kg]	<2.89	<0.60	x
BbF [ug/kg]	5.88 +/-2.32	12.49 +/-7.88	x
BkF [ug/kg]	3.03 +/-0.52	x	x
BPe [ug/kg]	<2.97	<0.73	x
Chr [ug/kg]	5.07 +/-1.55	<0.41	x
DahA [ug/kg]	<2.95	<0.61	x
Fla [ug/kg]	x	x	x
Flu [ug/kg]	19.86 +/-11.51	x	x
IPy [ug/kg]	x	<0.82	x
Nap [ug/kg]	<4.12	x	<6.10
Phe [ug/kg]	x	x	x
Pyr [ug/kg]	8.87 +/-6.92	56.73 +/-46.47	x

BLV-Nr.	18-01046-A	18-01046-C	18-01046-F
METAS-Nr.	200044720	200044721	200044719
Ace [ug/kg]	9.54 +/-6.08	<0.64	<7.10
Acy [ug/kg]	<3.00	0.50 +/-0.11	<3.88
Ant [ug/kg]	4.32 +/-1.41	<0.49	9.54 +/-6.90
BaA [ug/kg]	3.94 +/-1.05	<0.70	4.67 +/-1.70
BaP [ug/kg]	<2.72	<0.57	5.14 +/-2.27
BbF [ug/kg]	<2.69	0.63 +/-0.20	4.59 +/-1.76
BkF [ug/kg]	<2.59	0.64 +/-0.27	<2.92
BPe [ug/kg]	x	1.02 +/-0.57	<4.21
Chr [ug/kg]	6.29 +/-3.37	<0.40	6.10 +/-2.72
DahA [ug/kg]	x	<0.55	<3.54
Fla [ug/kg]	x	x	x
Flu [ug/kg]	16.14 +/-8.42	x	<5.45
IPy [ug/kg]	x	<0.75	<3.82
Nap [ug/kg]	19.86 +/-18.45	43.87 +/-40.52	<5.73
Phe [ug/kg]	x	x	x
Pyr [ug/kg]	x	32.28 +/-23.44	x

BLV-Nr.	18-01042-A	18-01042-C	18-01042-F
METAS-Nr.	200044716	200044717	200044715
Ace [ug/kg]	<4.47	x	<5.91
Acy [ug/kg]	8.99 +/-4.73	x	8.60 +/-4.87
Ant [ug/kg]	x	x	x
BaA [ug/kg]	9.08 +/-4.99	<0.71	3.83 +/-1.11
BaP [ug/kg]	5.47 +/-2.17	<0.59	<3.10
BbF [ug/kg]	<2.87	0.61 +/-0.34	<2.94
BkF [ug/kg]	<2.74	0.88 +/-0.44	3.02 +/-0.70
BPe [ug/kg]	4.97 +/-1.75	1.24 +/-0.71	<4.10
Chr [ug/kg]	<4.31	<0.41	6.65 +/-3.14
DahA [ug/kg]	<3.09	<0.58	<3.35
Fla [ug/kg]	x	x	x
Flu [ug/kg]	18.51 +/-10.51	x	17.65 +/-12.63
IPy [ug/kg]	<2.73	<0.80	<4.16
Nap [ug/kg]	x	x	x
Phe [ug/kg]	x	x	x
Pyr [ug/kg]	x	35.50 +/-26.48	13.82 +/-12.63

BLV-Nr.	18-01051-A	18-01051-C	18-01051-F
METAS-Nr.	200044724	200044725	200044723
Ace [ug/kg]	<4.04	<1.01	x
Acy [ug/kg]	5.77 +/-2.36	<0.50	<4.14
Ant [ug/kg]	7.84 +/-4.59	<0.49	x
BaA [ug/kg]	2.95 +/-0.59	<0.67	8.46 +/-4.81
BaP [ug/kg]	<2.66	<0.54	<3.37
BbF [ug/kg]	<2.56	<0.53	<3.19
BkF [ug/kg]	<2.47	<0.77	<3.08
BPe [ug/kg]	<2.99	x	<4.36
Chr [ug/kg]	4.30 +/-1.30	<0.39	12.14 +/-9.20
DahA [ug/kg]	<2.80	<0.54	<3.65
Fla [ug/kg]	x	x	x
Flu [ug/kg]	16.54 +/-9.62	x	x
IPy [ug/kg]	<2.45	x	x
Nap [ug/kg]	<3.80	x	<7.78
Phe [ug/kg]	x	x	x
Pyr [ug/kg]	x	19.42 +/-12.48	13.36 +/-11.74

BLV-Nr.	18-01043-A	18-01043-C	18-01043-F
METAS-Nr.	200045358	200045359	200045357
Ace [ug/kg]	17.33 +/-6.06	<6.19	12.31 +/-6.59
Acy [ug/kg]	15.98 +/-4.67	x	10.54 +/-2.76
Ant [ug/kg]	15.42 +/-5.47	<2.28	x
BaA [ug/kg]	17.11 +/-8.12	x	x
BaP [ug/kg]	x	<1.30	x
BbF [ug/kg]	x	x	x
BkF [ug/kg]	<2.34	x	x
BPe [ug/kg]	x	x	x
Chr [ug/kg]	x	x	x
DahA [ug/kg]	x	x	x
Fla [ug/kg]	x	x	x
Flu [ug/kg]	x	16.56 +/-4.37	x
IPy [ug/kg]	x	x	x
Nap [ug/kg]	<7.57	x	x
Phe [ug/kg]	x	8.57 +/-5.56	x
Pyr [ug/kg]	10.59 +/-5.99	12.88 +/-3.82	15.31 +/-7.96

BLV-Nr.	18-01047-A	18-01047-C	18-01047-F
METAS-Nr.	200045361	200045362	200045360
Ace [ug/kg]	18.47 +/-7.26	x	x
Acy [ug/kg]	9.38 +/-2.13	18.65 +/-8.70	x
Ant [ug/kg]	x	18.61 +/-12.26	x
BaA [ug/kg]	x	7.07 +/-1.35	3.45 +/-0.44
BaP [ug/kg]	x	x	<1.42
BbF [ug/kg]	x	x	x
BkF [ug/kg]	<2.26	5.83 +/-0.55	x
BPe [ug/kg]	x	x	x
Chr [ug/kg]	<1.49	4.37 +/-0.57	4.76 +/-0.64
DahA [ug/kg]	x	x	x
Fla [ug/kg]	12.24 +/-3.60	x	17.56 +/-4.56
Flu [ug/kg]	14.20 +/-12.55	x	18.18 +/-5.20
IPy [ug/kg]	x	x	x
Nap [ug/kg]	<7.17	x	x
Phe [ug/kg]	14.19 +/-8.41	18.02 +/-9.79	5.50 +/-3.63
Pyr [ug/kg]	<1.83	6.89 +/-4.06	0.67 +/-0.42
